# Evaluating Consumer Perceptions and Safety of Genetically Modified Foods in Africa: A Comprehensive Review

**DOI:** 10.1002/fsn3.4730

**Published:** 2025-06-03

**Authors:** Adeola Omotosha Dolapo, Helen Onyeaka, Chiemerie Theresa Ekwueme, Esther Ibe Njoagwuani, Olaoluwa Olowe Ayomikun, Chidinma Ezinne Ngene, Comfort Adeola Olatunji, Iyiola Oladunjoye, Ifeanyi Mazi Michael, Hope Akegbe, Phemelo Tamasiga, Soumya Ghosh

**Affiliations:** ^1^ Department of Cell Biology and Genetics, Faculty of Life Sciences University of Lagos Akoka Nigeria; ^2^ School of Chemical Engineering University of Birmingham Birmingham UK; ^3^ Department of Microbiology Federal University Otuoke Bayelsa Nigeria; ^4^ Department of Microbiology, Faculty of Life Sciences University of Benin Benin Nigeria; ^5^ Department of Microbiology Babcock University Ilishan‐Remo Nigeria; ^6^ Department of Food Science and Technology, Faculty of Agriculture University of Nigeria Nsukka Nigeria; ^7^ Department of Microbiology University of Ilorin Ilorin Nigeria; ^8^ CRETEGI, Centre of Research in Energy, Trade and Green Industrialisation Gaborone Botswana; ^9^ Natural and Medical Sciences Research Center University of Nizwa Nizwa Oman

**Keywords:** Africa, consumers, food insecurity, food safety, gene technology, genetically modified foods

## Abstract

Technological advances in the food industry have garnered significant attention, particularly for enhancing sustainability and reducing food insecurity in Africa, especially in sub‐Saharan regions where several million people are undernourished. This increasing demand for food, coupled with consumers' desire for diversity and safety, has spurred innovations like Genetically Modified Foods (GMF). These programs involve altering the genetic makeup of crops and livestock to improve outcomes such as yield, resistance to pests, and overall efficiency. GMF programs constitute several innovative strategies such as technological interventions, including the use of better seeds and pest control methods to combat poor harvests and inefficiencies. Transgenic technology, a key innovation, modifies specific genes in crops to enhance resistance to insects, pathogens, and environmental stressors, leading to increased productivity and efficiency for farmers. Despite these benefits, GMF has sparked public concerns and controversies related to ethics, potential increases in global antibiotic resistance, nutritional quality, toxicity, allergies, and environmental risks. While these issues have generated debate, most studies suggest that the advantages of GMF, such as improved crop quality and resilience, outweigh the disadvantages. This review highlights the safety of GMF, noting that natural gene recombination through selection, breeding, and mutation has long been beneficial. Additionally, it addresses consumer perceptions and the significant concerns surrounding environmental risks and health hazards associated with GMF. Ultimately, while GMF presents promising solutions to food insecurity and agricultural inefficiencies, ongoing research and dialogue are essential to address the ethical and safety concerns raised by the public.

## Graphical abstract

The manuscript highlights the potential of GMF to address food insecurity in Africa by improving crop yields, enhancing nutritional value, and reducing reliance on agrochemicals. However, there are public concerns about safety, environmental risks, and ethical implications, coupled with misinformation and inadequate regulatory frameworks that hinder their acceptance. The study emphasizes the need for robust safety assessments, public education, and inclusive policies to facilitate the adoption and sustainable use of GMFs in Africa.

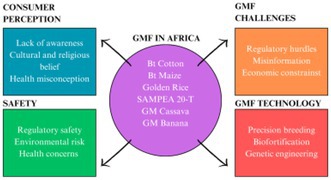

## 1 Introduction

The food industry has faced major setbacks in meeting the food demand of consumers over the years. Particularly, Africa faces a major setback in food production due to inherent factors of her environment. According to the Food and Agriculture Organization's (FAO) Africa Regional Overview of Food Security and Nutrition in 2023, the prevalence of undernourishment indicates that the burden of hunger in Africa is worsened with 19.7% of the population being undernourished in 2022, with 282 million (which makes up 38% of the estimated 735 million people) facing hunger globally. Additionally, the effect of hunger and undernourishment was reported to have increased over all regions in Africa particularly in Eastern Africa (FAO et al. 2023). This report shows a massive increase in the state of hunger compared to the reports of 2017 and 2018, before the COVID‐19 pandemic which showed 256 million people living in hunger (FAO et al. 2020). The highest inter‐annual report of undernourishment was reported in 2019 and 2020 coinciding with the onset of COVID‐19 (FAO et al. 2023). A recent estimate of food insecurity in Africa shows that 61% of the population faced moderate to severe food insecurity due to their inability to access enough food for the year; this statistic is twice the prevalence of 29.6% faced globally, with an increase of 74 million people between 2020 and 2021, with western Africa being the most affected in sub‐regional level (FAO et al. 2023). Associated health issues particularly among children between the ages of 1 and 5 years old have been massively reported. This hunger has been largely attributed to the inability to afford food, with reports showing that 77.5% of the population in Africa (over 1 billion people) will be unable to afford a healthy diet in 2021. This increased from 51 million people in 2019 (Bain et al. 2013). Generally, food insecurity and hunger in Africa have been attributed to a worsening shortfall in the production of food to meet the food demand of the growing population, and the unaffordability can be linked to the agricultural intensification with higher inputs, thereby increasing the cost of food. Each year, Africa loses half of its harvest to pests (insects, nematodes, pathogens, and weeds), poor storage conditions, and weather challenges, which impact crop production negatively and primarily increase malnourishment (IPCC 2022). This has kept Africa's food production rate slow relative to her population increase. Globally, most regions of the world have experienced an increase since 1970 in per capita food output, while Africa stands as an exception, far outside the balanced radius (Gbashi et al. 2021). Owing to the continent bearing the highest burden of foodborne diseases in the world, investigating and developing prepared responses to the threats of food insecurity is crucial.

These recent statistics on food challenges faced in Africa are only a reflection of previous adverse reports that had gradually worsened. Extreme hunger and malnutrition have remained a focus of the United Nations (UN) and a sustainable development goal (SDG) (Wudil et al. 2022). Over the years, Africa has relied largely on cross‐breeding as the sole means of acquiring desired traits and phenotypes among plants and animals (Gbadegesin et al. 2022); however, this has not been effective in eradicating the hunger surge. Today, genetic engineering has been able to speed up this grossly slow method of acquisition of traits in food production. The adoption and implementation of agricultural and food technologies has been considered integral in meeting the “Zero hunger” SDG “goal” which aims to eradicate hunger and increase sustainable agriculture (FAO 2021). Farmers have adopted various methods to improve crop production; this includes the use of fertilizers, pesticides, natural selection, inter‐breeding, crop rotation, mechanization, and improved seed varieties (Jacquet et al. 2022). In the same vein, scientists have been working on novel strategies such as precision agriculture, integrated pest management (IPM), integrated farming systems (IFS), and climate‐smart agriculture (CSA) to improve agricultural productivity and promote sustainable food production (Muhie 2022). Biotechnological advances have been used in the production of food crops that are more resistant to harm from common food crop diseases and spoilage pathogens. It further decreases the need for the use of chemical pesticides and insecticides that affect the environment among other beneficial characteristics (Gbashi et al. 2021; James 2013). Genetically modified (GM) foods are foods from animal or plant sources whose deoxyribonucleic acid (DNA) has been modified from its natural form using recombination DNA technology to produce foods or food ingredients with targeted quality characteristics, such as increased yield, disease resistance, added and enhanced nutritional value, and longer shelf life among others (FSA 2018; Bawa and Anilakumar 2012). GM crops have been widely accepted and grown in economically significant amounts in millions of hectares in the United States, Brazil, India, Canada, and Argentina with Soybean, maize, cotton, and canola being the most adopted commercialized crops (Reidy 2023; ISAA 2018). In Africa, South Africa commercialized GM maize in 1988 with *Bacillus thuringiensis* (Bt) maize (Monsanto 810) and pest‐resistant Bt11 in 2003 (Abidoye and Mabaya 2014). Due to the promising future of this technology to meet the estimated 50% increase in food production and to meet the global nutritional need (FAO et al. 2017), many African countries have adopted biosafety laws and formulated standardized regulatory guidelines through the government and national decision‐making bodies to regulate GM activities and ensure safety (Gbadegesin et al. 2022; Akinbo et al. 2021). Despite efforts to utilize the benefits of GMF, government policies, and internationally harmonized scientific approaches to risk assessment and regulation frameworks, there have been setbacks in the global acceptance of GMF (Smyth et al. 2021). Only five African countries (South Africa, Burkina Faso, Malawi, Egypt, and Sudan) farm GM crops. Furthermore, only South Africa, Zimbabwe, Kenya, Sudan, Egypt, Tunisia, Algeria, Mali, Burkina Faso, Togo, Ghana, and Mauritius have operational National Biosafety Frameworks (NBFs) set in place (Akinbo et al. 2021).

Globally, there has been a notable breakthrough in the food industry with the adoption of GMF; GM rice called the “golden rice” engineered to produce high levels of β‐carotene to combat vitamin A deficiency (VAD) in children, which has affected over 250 million children, has been evaluated with the capacity to produce 99% of VAD requirement relative to conventionally produced rice (Wu et al. 2021; Swamy et al. 2019). However, the commercialization of GMF has been constrained due to concerns raised in line with health hazards, environmental risks, and consumer perception. Consumer perception of GMF in Africa has been regarded as a chief constraint in the acceptance of GMF, even though they could arguably benefit the most from GMF. In a study conducted in Abuja, Nigeria, on consumer perception of GMF, Iroh et al. (2021) reported that a whopping 67.46% of the respondents opined that GMF is harmful to human health, 51.98% linked GMF to high allergic reaction, 18.25% perceive GMF to be toxic, 12.4% believed that GMF are linked to negative environmental impact, and 49.6% are concerned about the long‐term effect of GMF. Similar consumer perception trends have been reported in Tanzania (Mnaranara, Zhang, and Wang 2017), Kenya (Kagai 2011), Uganda (Mustafa et al. 2023), Ghana (Deffor 2014), and Zimbabwe (Dexter et al. 2019). Global trends and the need to eradicate hunger and undernourishment are advancing the need for GMF technology and innovation. However, poor implementation of innovation and technological strategies in GM farming and antibiotic resistance among livestock can lead to unsustainable agricultural practices that can further lead to loss of biodiversity, environmental risk, and harmful health hazards among consumers (Masud et al. 2020).

## 2 Hunger and Genetically Modified (GM) Technology in Africa

**FIGURE 1:**
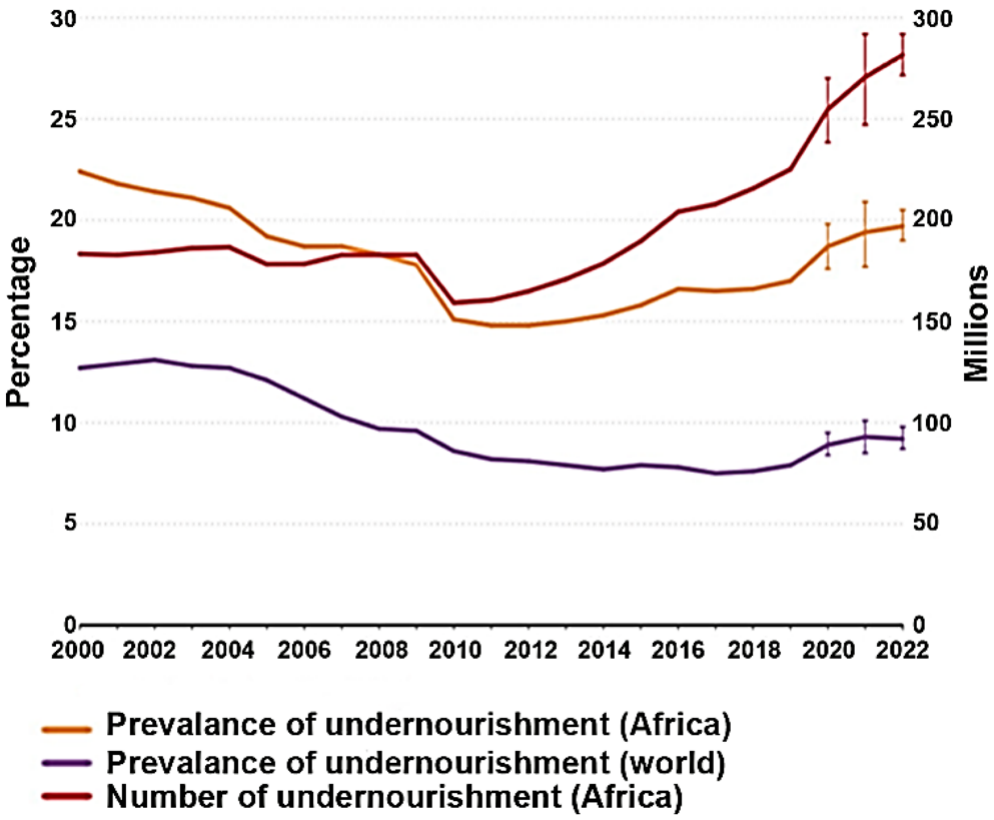
Prevalence of undernourishment in Africa, the world, and the number of undernourished people in Africa (FAO et al. 2023).

Amid several global issues, Africa continues to face deepening food‐related challenges. Thirty‐eight percent of Africa's population is saddled with the daily challenge of hunger, making up 282 million hungry people of the 735 million people that are faced with hunger globally; 61% of Africa's population are faced with moderate to severe food insecurity due to inability to obtain adequate food through the course of a year, straining the SDGs due to the high level of malnutrition, particularly stunting among children under 5 years of age in 2022 (FAO et al. 2023) (Figure 1). Increased levels of hunger and malnutrition have been a long age issue in Africa, with statistical evidence before the COVID‐19 pandemic proving that the concerns only worsened due to the pandemic. Undernourishment prevalence in Africa between 2005 and 2017 consistently exceeded the global prevalence with significant statistic gaps, which reduces the effect of short‐lived and temporary crises on the problem of elevated hunger in Africa (FAO and ECA 2018). Africa's population poses a considerable amount of pressure on the food production industry due to the high food demand. The boost in food prices, livestock mortality, and diminishing crop yield and harvest were the aftermath of increased cases of diseases and pests in Africa with Southern and Eastern Africa bearing the highest burden of food insecurity (Otekunrin et al. 2020). Africa's agricultural system is vulnerable to climate change and its associated hazards such as floods, erosion, pests, droughts, and diseases, which reduce the amount of produce and poor development of seeds and require high input from farmers (Tambo et al. 2023; Sirba and Chimdessa 2021). This system is further constrained by the challenges of shifting to sustainable options while producing safer foods. The shift to sustainable farming practices birthed the need for innovation and novel food technologies, particularly biotechnology (Bhat 2022).

**FIGURE 2:**
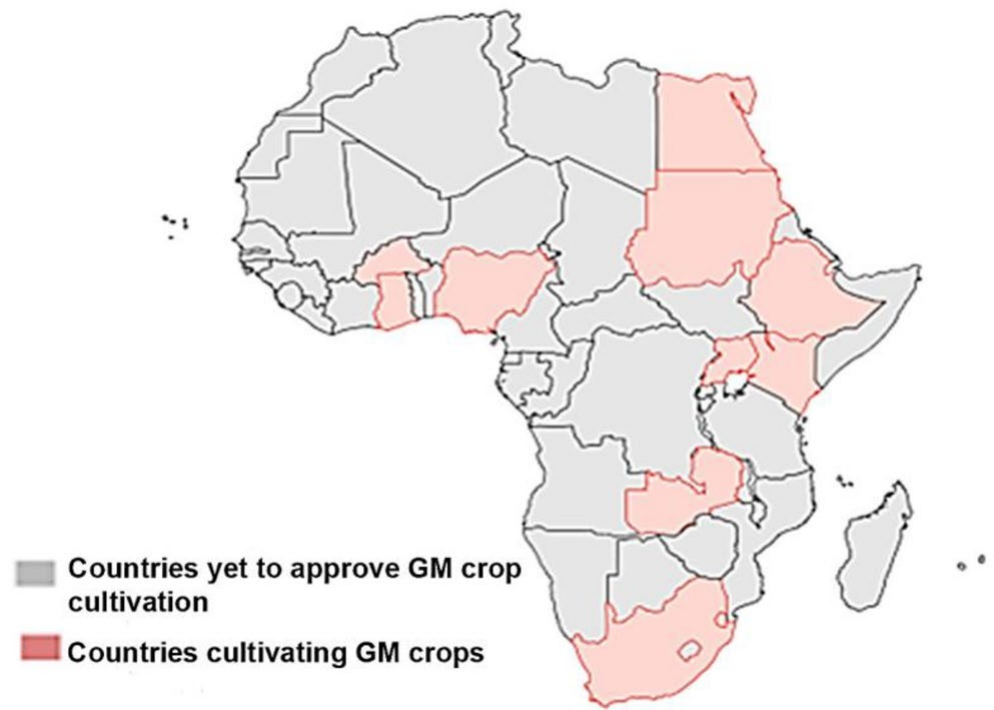
African countries' status of GM crops based on ISAA GM approval database in 2024 (Adopted from: https://www.isaaa.org/gmapprovaldatabasE) (Created using Bio Render).

Biotechnological development and advances have seen the birth of food crop yields with increased resistance to disease‐causing and food spoilage agents, which in turn has limited expense and reliance on pesticides and insecticides that pose risks to the environment. Regarding biotechnology, GM technology is considered a potential tool for achieving sustainable pathways in Africa (Oliver 2014). This technology has evolved from the method involving the editing of a plant's genome to make targeted improvements, within the plant's genome (Kaur et al. 2022), to the modification of a plant's genome involving the introduction of genetic materials from a different organism to produce a transgenic organism (Sarker et al. 2019), which differ from traditional breeding approaches adopted in Africa that have yielded relatively insignificant results. GM technology has led to significant pest and disease resistance among other beneficial characteristics (James 2013), which particularly addresses the major food production setbacks in Africa; however, there have been concerns surrounding the safety and sustainability of GMF, and this has led to several debates on the consumption, cultivation, and commercialization of GMF and has further led to restraint in the adoption of GM technology in Africa. The research, development, and deployment of biotechnological crops in African countries are in varying levels of evolution as crops for commercial purposes and farmers' usage are being deployed by a few countries (Akinbo et al. 2021) (Figure 2). South Africa holds the record as the biggest producer of genetically modified crops in Africa and is also the first country in Africa to establish the cultivation, importation, and exportation of genetically modified crops based on a regulatory framework (Endale et al. 2022).

Among 54 African nations, Ghana, Malawi, Sudan, Burkina Faso, Nigeria, Uganda, Egypt, South Africa, and Kenya are conducting GM crops field trials with commercialization only in Egypt, Burkina Faso, Sudan, and South Africa (Mabaya et al. 2015). The commercialization of GM crops and the introduction of Bt maize (Monstano 810) and pest‐resistant Bt11 in South Africa in 2003 ushered a new era in crop production (Abidoye and Mabaya 2014). This breakthrough was followed by the commercialization of GM crops in Egypt and the introduction and commercialization of Bt cotton in Burkina Faso in 2008. The development of biotechnology in Africa has led to a reduction in poor maize harvest and gains arising from damages caused by stem borers (ASSAF 2010). In 2019, Bt cotton was adopted in Kenya and pod‐borer‐resistant cowpea in Kenya. Ethiopia, Malawi, Sudan, and Eswatini have also adopted Bt cotton (ABNE 2022). Generally, about 3 million hectares account for the planted GM crops in Africa (Gbashi et al. 2021; Azadi et al. 2015). In 2019, in Nigeria, two GM crops—cowpea (pod‐borer‐resistant cowpea named SAMPEA 20‐T) and pest‐resistant Bt cotton—indicated a major progression in agricultural technology (Boluwade and Uche 2021). The adoption of GM bananas by Tanzanian farmers in 2011 has proven effective in meeting food demands due to their resistance to Xanthomonas wilt (Kikulwe, Wesseler, and Falck‐Zepeda 2011). Ghana considered an emerging adopter of GM technology in crop production is set to approve commercial cultivation of GM crops based on evidence‐based decisions (Turnbull, Lillemo, and Hvoslef‐Eide 2021; Komen et al. 2020). Some prominent heads of state are actively supporting GMF. Senegalese President Macky Sall approved a new biosafety legislation in 2022, thereby repealing the previous biosecurity law from 2009 and allowing Senegal to fully maximize the contemporary advantage of biotechnology. Dr. Joyce Banda, former Malawian president, also made notable steps in incorporating GM crops into farming, creating room for Malawi to go into the research of GM crops like maize, cassava, cowpea, and beans. Ethiopia among others also ensured the approval of the commercial cultivation of cotton and maize as an avenue to improve agricultural productivity in 2018 (Maina 2022). Countries where GMOs have been cultivated and commercialized in Africa grew from 3 to 10 within the years 2016–2022 (Oloo 2022). Despite the global trends, Kenya has been slow to make full‐fledged use of GMF due to concerns about the health and safety of GMF (Abidoye and Mabaya 2014). This was due to a seven‐year ban on GM crops (Gbashi et al. 2021), which has been lifted and ushered in the transition to Bt cotton in 2020. In 2021, the Tanzanian agriculture minister banned research trials requiring the use of GM crops (Mmbando 2023); this stand was further strengthened by the new minister in 2022, with a ban on the importation of GM crops (Anami and Bob 2022) Tanzania is behind Kenya and Uganda with high global hunger index (GHI) and GM technology‐related research (Lewis et al. 2010). The Uganda government is yet to set regulations that will govern the cultivation and commercialization of GM crops in Uganda (Lukanda 2020). Generally, the cultivation and commercialization of GMF in African countries have been adopted by only a small group of African nations (Table 1), which poses a restraint in the eradication of hunger using biotechnology. The standpoint in accepting GMOs by African nations could have been a result of several internal and external factors such as lack of individual knowledge and awareness of GMOs, knowledge of genetic engineering/biotechnology, poor evidence for health issues claims as in the case of Kenya, knowledge gap of the modern implementation of biotechnology, absence of regulatory policies, poor sensitization/awareness, and misinformation (Sefater 2021).

**TABLE 1:** Some GM crops cultivated and/or commercialized by African countries and the modified genetic traits conferred on the crops.

Crop	Country	Genetic traits	References
Sudan	Cotton	Insect‐resistant varieties with Bayer's Bollgard trait	USDA (2019)
Ethiopia	Cotton	Insect‐resistant	Gebre et al. (2023)
Kenya	Cotton	Insect‐resistant	ISAAA (2020)
	Sweet potatoes	Virus resistance, late blight resistance	GM Monitor (2023)
	Cassava	Cassava mosaic and brown streak resistance	USDA (2023a)
	Sorghum	Nutrient density	Ehirim et al. (2020)
	Maize	Water efficient maize	Schnurr and Dowd‐Uribe (2021)
Nigeria	Maize	Drought‐tolerant and insect‐resistant	
	Cowpea (Sampea 20‐T)	Resistant microbial insecticidal traits, increased histidine, isoleucine, and leucine	Barrero and Higgins (2020), Amedu et al. (2023)
	Cassava	Elevated levels of zinc and iron, brown steak disease resistance	USDA (2023a)
Uganda	Banana	Banana *Xanthomonas* wilts resistant	Namukwaya et al. (2011)
	Sweet potatoes	Virus resistance	
	Cassava	Brown steak disease resistance	USDA (2023a)
	Maize	Insect‐resistant, drought‐resistant	Ehirim et al. (2020)
	Soybean	Herbicide‐tolerant	
	Rice	Nitrogen‐efficient, water‐efficient, and salt tolerant	Ehirim et al. (2020)
Malawi	Cotton	Insect resistance	Ehirim et al. (2020)
South Africa	Rice	Herbicide‐tolerant	ABNE (2019)
	Cotton	Insect‐resistant and herbicide‐tolerant	ABNE (2019)
	Canola	Herbicide‐tolerant	ABNE (2019)
	Sugarcane	Sugar content, virus‐resistant	Ehirim et al. (2020)
	Soybean	Abiotic stress tolerance, herbicide tolerance	OECD (2021)
	Maize	Insect‐resistant and herbicide‐tolerant	USDA (2019), Mukarumbwa and Taruvinga (2023)
Eswatini	Cotton	Resistance against pest infestation	Khumalo and Bimha (2018), Lewis, Masinjila, and Sirinathsinghji (2017)
Ghana	Cowpea	Insect‐resistant	Addae et al. (2020)
	Coconut	Virus‐resistant	Ehirim et al. (2020)
Egypt	Maize		
	Potatoes	Virus, pest, and fungal resistant	Ehirim et al. (2020)
Ethiopia	Cotton	Resistant against pest infestation	Kedisso et al. (2023)
Burkina Faso^a^	Cotton	Insect‐resistant	Luna (2020)
Tanzania	Cotton	Insect‐resistant	Ehirim et al. (2020)
	Maize	Drought‐resistant, insect‐resistant	Ehirim et al. (2020)
	Cassava	Cassava mosaic disease (CMD) and cassava brown streak disease (CBSD)‐resistant, drought‐resistant	Nyinondi et al. (2017)

*Note:* Reports are available on the ISAA GM approval database.

^a^ Production of GM was reportedly halted in 2015 (Dowd‐Uribe and Schnurr 2016).

## 3 Impact of GMFs on Food Security in Africa

One of the most direct impacts of GMFs on food security in Africa is through the enhancement of agricultural productivity. Traditional farming methods in many African countries have been hampered by various factors such as poor soil fertility, unpredictable weather patterns, and the prevalence of pests and diseases. GMFs, designed to be more resistant to these challenges, can significantly improve crop yields (Sadikiel Mmbando 2024). A notable example of success is seen in South Africa, where the commercial production of insect (such as stem borer)‐resistant *B. thuringiensis* (Bt) *Zea mays* L. and *Gossypium hirsutum* L. has significantly increased yields and reduced the need for chemical pesticides (Gouse 2012; Muzhinji and Ntuli 2021; Cooper 2022). Higher yields directly contribute to food security by increasing the availability of food at both local and national levels. In addition to boosting crop yields, GMFs can also help in reducing post‐harvest losses, a significant issue in Africa where up to 30% of harvested crops can be lost due to factors such as pests, diseases, and poor storage conditions (Akpa et al. 2023). For instance, post‐harvest losses of groundnuts in African countries range from 8.9% in Ghana to 31% in Uganda. Despite the importance of quality, producers of higher‐quality groundnuts do not receive better market prices because traders and processing factories typically do not test for aflatoxin content during purchase (Daba et al. 2023). Therefore, using GM crops that are engineered for longer shelf life or resistance to aflatoxin spoilage can reduce these losses, ensuring that more of the food produced reaches consumers. This not only improves food availability but also helps stabilize prices, making food more affordable for the population (Sadikiel Mmbando 2024). Another critical aspect of food security is nutrition. In many African countries, the diet is often limited in diversity, leading to deficiencies in essential nutrients like vitamin A, iron, and zinc (Grabowski et al. 2024). Biofortified GMFs, which are crops genetically enhanced to contain higher levels of these nutrients, offer a promising solution. For instance, Golden Rice, which is fortified with beta‐carotene (a precursor of vitamin A) (Nguyen et al. 2021), has the potential to address vitamin A deficiency in regions where rice is a staple food (Paine et al. 2005). Therefore, by improving the nutritional content of staple crops, GMFs can help alleviate malnutrition and improve the overall health of populations.

The economic impact of GMFs on smallholder farmers, who constitute a significant portion of Africa's agricultural workforce, is another important consideration. GM crops, with their higher yields and resistance to pests and diseases, can reduce the need for costly inputs like pesticides and fertilizers, thereby lowering production costs (Klümper and Qaim 2014). This can increase the profitability of farming, providing farmers with more income to reinvest in their operations, purchase more food, and improve their living standards. However, it is important to note that the adoption of GMFs also requires access to capital, knowledge, and infrastructure, which may not be readily available to all farmers, particularly in less developed regions (Sadikiel Mmbando 2024).

Despite the potential benefits, the adoption of GMFs in Africa has been met with resistance and controversy. Concerns about the long‐term environmental impacts of GM crops, such as the potential for gene flow to non‐GM crops and the development of pest resistance, have been raised. Additionally, issues related to intellectual property rights and the control of seed markets by a few large corporations have led to fears of increased dependency and reduced sovereignty for African nations (Moore 2013). These challenges need to be carefully managed to ensure that the benefits of GMFs can be realized without exacerbating existing inequalities or environmental problems. Therefore, GMFs have the potential to significantly enhance food security in Africa by improving agricultural productivity, reducing post‐harvest losses, and addressing nutritional deficiencies. However, realizing these benefits requires careful consideration of the economic, environmental, and social challenges associated with GMFs. A balanced approach that includes robust regulatory frameworks, public awareness campaigns, and support for smallholder farmers will be essential for ensuring that GMF contributes positively to food security in Africa.

## 4 Sources and Production of GMF for African Consumption

Globally, 11 of 71 countries that issued regulatory approval for GM crops commercial cultivation were African countries with a total of 4349 total approvals between 1992 and 2018 marking a 113‐fold increase (Ichim 2020). In 2019, four countries in Africa were commercially approved for GM crops: Ethiopia (Bt Cotton), Malawi (Bt Cotton), Kenya (Bt Cotton), and Nigeria (PBR cowpea) for initial cultivation (ISAAA 2020). In 2017, a total of 190 million hectares was reported as the total global acreage of GM crops cultivations with African countries having a share of 3 million hectares with 2.7 million reportedly grown by South Africa alone followed by Sudan with 192,000 ha (Endale et al. 2022). In 2021 Nigeria approved TELA maize (Zambrano et al. 2022; Premium Times 2021), and in 2021, Kenya approved GM cassava (Ntui et al. 2023; USDA 2023a). In Africa, the cultivation and commercialization of some GM crops have been reported (Table 1). These reports show that GM crops have been approved and are successfully cultivated and commercialized for consumption in Africa.

However, smallholder agriculture is predominant and undoubtedly suffers from a lack of agricultural investment to meet the need for GM cultivation and commercialization due to low budgetary allocation. In recent years, several multilateral organizations have begun to research GM crop technologies for poverty reduction, economic growth, food security, and development. This has promoted public‐private partnerships (PPPs) that are focused on pooling finances and mitigating certain associated risks in food production networks. In Africa, certain PPPs are primarily focused on the research and development of GM foods through various sponsored projects (Table 2). This involves activities of several stakeholders such as private sectors (agro‐dealers, seed companies, and research institutes), public sectors, and non‐governmental organizations (NGOs). They spearhead research on certain GM crops, make available pre‐commercialized hybrid seeds, and pass these through seed companies for national performance trials (NPTs); foundation seeds are further produced and made available to farmers through agro‐dealers (Gebre et al. 2023). A typical example of a project that sponsors GM foods in Africa is the Water Efficient Maize for Africa (WEMA) currently funded by the Bill and Melinda Gates Foundation, Howard G. Buffet Foundation, and the United States International Agency for Development (USAID). This project is aimed at providing drought‐resistant maize seeds to farmers to reduce the incidence of low productivity in moderate drought as this affects food production in Africa and impacts yields (ACBio 2022).

**TABLE 2:** Public‐private partnership (PPP) programs targeted at improving adoption of GM crops in Africa.

PPP projects	Public‐private partners	Participating countries	Goals	References
Water Efficient Maize for Africa (WEMA) (2008)	Kenya Agriculture and Livestock Research Organization (KALRO) Mozambican Agrarian Research Institute (IIAM) South African Agricultural; Research Council (ARC) Tanzanian Commission for Science and Technology (COSTECH) Ugandan National Agriculture Research Organization (NARO)	Kenya, Mozambique, South Africa, Tanzania, and Uganda	To develop and disseminate drought‐tolerant and insect‐resistant maize to African farmers using GM technology, and conventional breeding	ACBio (2022)
Drought Tolerant Maize for Africa (DTMA) (2006)	International Maize and Wheat Improvement Center (CIMMYT) International Institute of Tropical Agriculture (IITA)	Angola, Benin, Ethiopia, Ghana, Kenya, Malawi, Mali, Mozambique, Nigeria, Tanzania, Uganda, Zambia, Zimbabwe	To mitigate drought and other problems relating to the cultivation of maize and increasing maize yields by 20%–30% under moderate drought in 13 African countries	CIMMYT (2018)
Insect Resistance Maize for Africa (IRMA) (1999)	International Maize and Wheat Improvement Center (CIMMYT) Kenya Agricultural Research Institute (KARI)	Kenya	To improve cultivation of maize and food security by the reduction of losses due to stem borers	Tefera, Mugo, and Beyene (2016)
Blight‐Resistant Potatoes	National Root Crops Research Institute (NRCRI) African Agriculture Technology Foundation (AATF)	Nigeria	To develop a variety of blight‐resistant potatoes	USDA (2022)
Accelerating Genetic Gains in Maize and Wheat Improvement Livelihoods (AGG) (2020)	International Maize and Wheat Improvement Center (CIMMYT) International Institute of Tropical Agriculture (IITA)	Benin, Ethiopia, Ghana, Kenya, Malawi, Mali, Mozambique, Nigeria, South Africa, Tanzania, Uganda, Zambia, Zimbabwe	To accelerate the development of higher‐yielding maize and wheat	CIMMYT (2020)
Stress Tolerant Maize for Africa (STMA) (2015)	International Maize and Wheat Improvement Center (CIMMYT)	Benin, Ethiopia, Ghana, Kenya, Malawi, Mali, Mozambique, Nigeria, Tanzania, Uganda, Zambia, Zimbabwe	Improve multiple stress‐tolerant maize varieties to address the problems of production and increased scale‐up	Kedisso et al. (2023)
Virus‐Resistant Cassava (VIRCA) Project (2016)	Donald Danforth Plant Science Centre The Kenya Agricultural and Livestock Research Organization The Uganda National Agricultural Research Organization (NARO)	Kenya, Nigeria, Uganda	To develop virus‐resistant and nutritionally improved cassava varieties in Africa	Robson, Hird, and Boa (2023)
Nitrogen‐Efficient, Water‐Efficient Salt‐Tolerant (NEWEST) Rice Program (2008)	United States International Agency for Development (USAID) African Agriculture Technology Foundation (AATF)	Ghana, Nigeria, Uganda	To develop and disseminate locally adapted rice varieties with improved nitrogen use efficiency, water use efficiency, and salt tolerance	AATF (2024)
The African Biofortified Sorghum (ABS) (2004)	Africa Harvest International (AHI) led consortium Bill and Melinda Gates Foundation	Burkina Faso, Ghana, Malawi, Nigeria	To develop high vitamin A, iron, and zinc‐containing sorghum	Dowd‐Uribe et al. (2023), Wambugu et al. (2015)
Pod Borer Resistant (PBR) Cowpea Program	United States International Agency for Development (USAID) African Agriculture Technology Foundation (AATF)	Burkina Faso, Ghana, Malawi, Nigeria	To increase productivity by developing PBR‐resistant cowpea	Dowd‐Uribe and Schnurr (2016)
Bio Cassava Plus Initiative	International Institute of Tropical Agriculture (IITA) Centro Internacional de Agricultura Tropical (CIAT)	Nigeria	To develop and introduce high‐yielding cassava varieties in Africa using breeding and biotechnology	Gbadegesin (2013)

Most African countries do not possess incentives for high‐end modern biotechnological farming and hence rely on the importation of food from other countries. Some GM crops and seeds are imported into Africa based on established guidelines for planting and food processing. In 2022, the National Biosafety Management Agency (NBMA) approved the importation of GM drought‐resistant wheat (HB4) from Argentina mainly for food and processing (USDA 2022). In East Africa, Kenya permits the importation of GM maize for human consumption (Mmbando 2023). As of 2015, Angola and Zimbabwe reportedly prohibit cultivation but approve the importation of GMF (James 2015). Additionally, South Africa and other countries of the world that cultivate and commercialize GM foods such as the United States occasionally donate food to African countries as relief aid during drought.

## 5 Food Safety and Need for GMF in Africa

The safety of food is a crucial component of a continent's health and development. Food safety encompasses all procedures that are adopted during the cultivation, harvesting, processing, and sale of food to prevent physical, chemical, and microbiological contamination that can lead to food‐borne diseases and affect consumer's health (Macieira, Barbosa, and Teixeira 2021). In Africa, there have been challenges related to food safety and management that have progressively worsened with the poor safety culture within its borders (Ayalew, Kareem, and Randolph 2023). Unsafe foods adversely impact public health and slow international agricultural trade further impacting the economy and development in Africa (Kareem, Martínez‐Zarzoso, and Brümmer 2022). The burden of foodborne illness is estimated to be the highest in Africa and South‐East Asia region with the highest incidence and morbidity (WHO 2001).

Food safety concerns are usually exacerbated by poor handling and contamination. Additionally, plant diseases and pests influence the safety of plants and affect their quality for human consumption (Savary et al. 2017). Food plants serve as a vehicle for harmful pathogens and microbial toxins that make food unsafe and cause food‐borne illnesses (Rizzo et al. 2021). Africa's food system is burdened with decreased productivity, largely due to climate change, which influences the persistence and prevalence of pests and microbial pathogens. Mafongoya et al. (2019) reported the transportation of destructive viral pathogens through pests in farmlands in South Africa, with reports showing begomoviruses, crinin, and tomato torrado virus (ToTV), in the study area. Aside from facilitating fungal growth and proliferation, the presence of insect pests in food crops is a food safety concern as insects are equally known to be carriers of foodborne pathogens (Pava‐Ripoll et al. 2015). The extent of pest‐associated damage to crops is expected to spiral given the effects of climate change all over the world (Ngumbi 2017). Additionally, corn has been marked to have high risk of aflatoxin contamination and proliferation facilitated by the increased temperature and humidity in the African region (Hell, Mutegi, and Fandohan 2010). Aflatoxins are a group of fungal toxins commonly *Aspergillus flavus* and *Aspergillus parasiticus*. Aflatoxins are known to contaminate crops such as corn, ground nuts, tree nuts, cottonseed, etc. during farming, harvesting, warehousing, or processing activities. Aflatoxin exposure causes liver failure and liver cancer. It is estimated that around 4.5 billion people residents in developing countries are exposed to aflatoxin contamination from home‐grown food crops due to poor farming, harvesting, warehousing, and processing methods and insufficient analytical testing (Hamid et al. 2013). About 40% of liver cancer and death cases in Kenya arise from acute aflatoxin poisoning (National Cancer Institute 2018). Furthermore, pesticides have been majorly adapted to combat pests and microorganisms that affect crops (Ramakrishnan et al. 2019). Pesticides are considered a great solution to compacting pests and pathogens in the food system; however, there is yet to be a balance between their potential and their possibility to cause harm, as reliance on pesticides threatens biodiversity loss (Raven and Wagner 2021; Zabel et al. 2019) and impacts food safety as the residues from pesticides and their metabolite can contaminate the food supply chain causing toxicity to human health. Akomea‐Frempong et al. (2017) reported the presence of certain metabolites from pesticides in high concentrations in ready‐to‐eat vegetables in Ghana. This result is similar to multiple pesticide residues reported in fruits and vegetables in Uganda (Ssemugabo et al. 2022), insecticide residues deposited in spinach, kaki, zucchini, and strawberry in Egypt (El‐Sheikh et al. 2022), and contamination in honey samples in Algeria (Bouhala et al. 2020). These reports explain the concerns of food safety related to the use of pesticides to combat the concerns of climate change and pests and diseases that burden food production in Africa. Pest‐ and pathogen‐resistant varieties are generally one of the most economical approaches to managing diseases on farmlands to minimize the occurrence of unsafe foods in Africa's food supply chain.

GM offers insect‐resistant crops with minimal damage, reduced fungal growth, and consequently a safer and healthier alternative to the use of chemicals (Gbashi et al. 2021; Gasperini et al. 2020). A meta‐analytic study of 147 food and feed crops reported a 37% decrease in the use of chemical pesticides and a 22% and 68% increase in crop yield and farmer's profit, respectively (Klümper and Qaim 2014). Cultivation of GM crop varieties has been noted as a valid pest control technology and a great option for controlling disease occurrences (Bawa and Anilakumar 2012; Waterfield and Zilberman 2012). These characteristics can serve as a solution to the problems facing the African harvest, by increasing the harvest per year to meet the growing food demand of the African population.

However, the debate surrounding the food safety of GM crops has consistently grown over the years and has primarily been associated with toxicity, food allergens, transfer of antibiotic resistance to humans, and the unknown long‐term effect on the environment. Just like all new technologies, GM technologies may pose some risks; nevertheless, no evidence proves that GM foods are unsafe to eat because of the GM technology used on the crop, and there is no evidence to prove that it is entirely safe either (Teferra 2021). Concerns relate to the modification of genes in food which may include the introduction of a different gene or editing of the normal genetic makeup of crops, and both processes may affect the long‐term expression of the genes which could lead to the production of new proteins or allergic reaction due to protein similarity with previously existing allergens. Additionally, concerns also spring up regarding the pest/weed resistant traits that are characteristic in GM crops. Just like antibiotic‐resistant bacteria, concerns have produced further debates regarding the resistance of these modifications by weeds and pests which may lead to reliance on GM technologies to further edit plant genomes causing a global burden of pest and weed resistance in food production that may worsen the present situation of food production today. Additionally, debate also surrounds the possibility of the formation of links between genes causing drug resistance in humans and gene flow in the natural drift and flow system that may cause the formation of ecological imbalance in the environment (Gu 2023). Some argue in response to this that conventional cross‐breeding and modification of genes have been traditionally adopted for decades which supports cross‐breeding, hybridization, and wide crosses, including inter‐species crossing, especially among perennial crops. However, this permits only the movement of extremely tiny fractions of genetic material; hence, one would expect that the product of this method will be stable and predictable unlike GM technologies that permit the movement of a high number of genetic materials (Martínez‐Fortún, Phillips, and Jones 2022). Hence, the possibility of food safety risks should not be ignored, and there should be an imposed and focused rational use of GM technology to minimize significant occurrences of accidents and harmful effects on the environment. Another source of concern is the issue of seed saving. Many schools of thought believe adopting GM crops can erode indigenous/traditional crops as witnessed in Mexico where surplus relief food (maize) was planted by farmers which eroded the natural varieties. Adopting GM crops may cause farmers not to save seeds as usually practiced (Ngaira and Ngaira 2017). This concern can be mitigated by properly managing indigenous varieties even while adopting GM crops, this ensures biodiversity and is able to lower overdependence on GM crops (Ngaira and Ngaira 2017). Labeling with appropriate communication of sources of food production to consumers and traceability of GM foods in food supply chain is also paramount as it imposes a legal tolerance threshold and informs consumer choice. Approaches to sidestep the unpredictability of GM technology should be adopted, especially as it relates to farmers' education and the cautious use of approved GM seeds according to the approved pattern of use for food safety proceeded by scientific testing and recommendation (Sadikiel Mmbando 2024).

## 6 Procedures for Approval of GMF Within Africa

**FIGURE 3:**
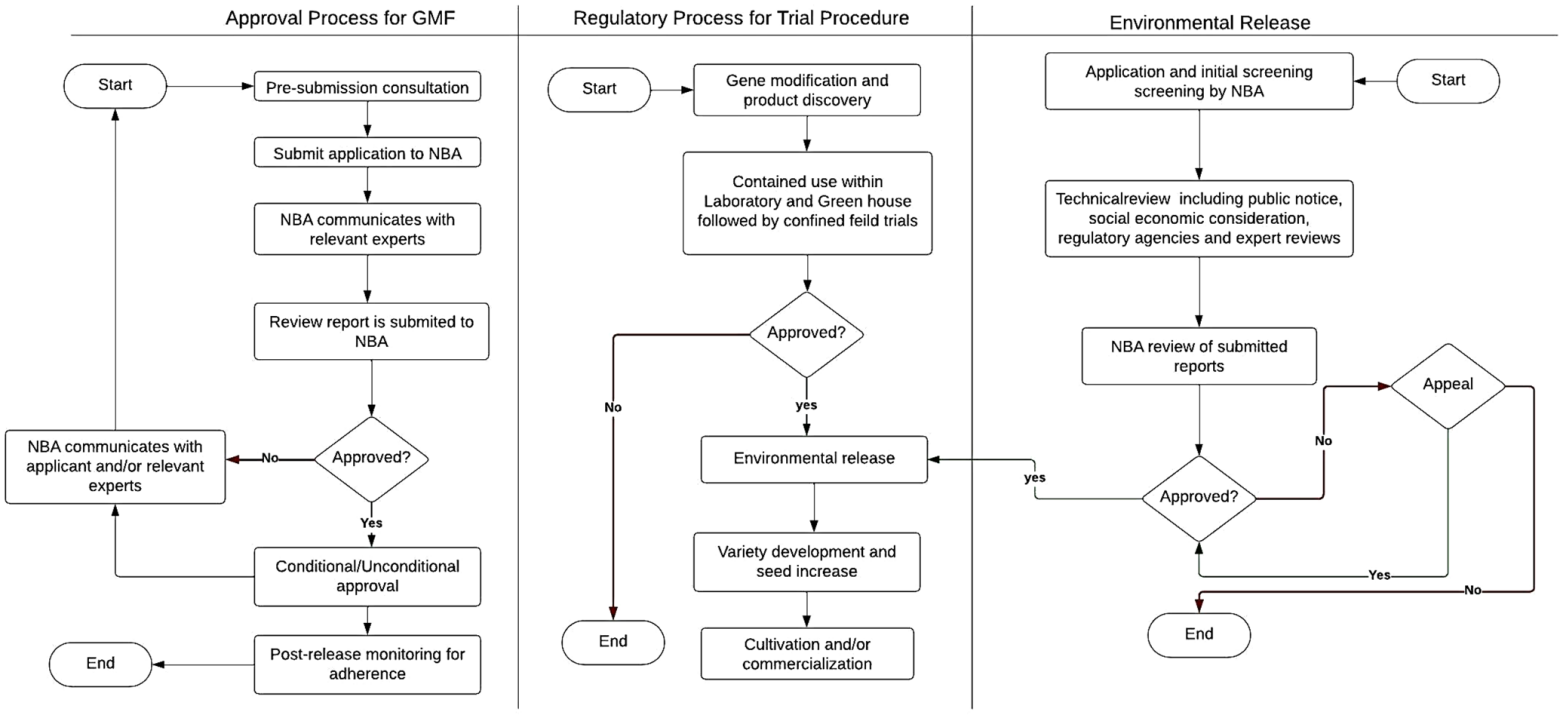
Procedure for the approval of genetically modified food in African countries (Adapted from Kenya NBA: https://www.biosafetykenya.go.ke/index.php?option=com_content&view=article&id=17&Itemid=122) (Created using Lucidchart).

In Africa, the biosafety regulatory framework of each country is mandated to approve the cultivation and/or commercialization of GM crops based on submitted data that has been examined and reported by several interfaces to ensure that GMF is safe for human consumption and environmental release. This procedure is based on a systematic approach that involves different regulatory bodies, experts, and the public (Figure 3). The Biosafety Authority is a designated government authority with the responsibility of receiving and reviewing submissions, coordinating the review process, and communicating feedback. Different African countries enacted biosafety acts and established biosafety authorities: the National Biosafety Authority (NBA) in Kenya, the National Biosafety Management Agency (NBMA) in Nigeria, and the National Biosafety Commission (NBC) in Uganda, among other African countries (Ongu et al. 2023). Upon receiving the application for approval of a GM plant/seed, a technical evaluation of the application is conducted by relevant experts including government authority and authorized regulatory bodies, the assessment seeks to understand the properties of the engineered trait and its effect on the test plant to assess its relevance to agricultural need of the country and potential risk, persistence, and dispersal in the environment; previous records of other similar release; the potential out‐crossing with other species; the effect on the flora, fauna, and non‐target organism; risk exposure; and risk management. Proper attention is accorded to relevant issues concerning new plants to avoid risk. During this consideration, regulatory approval for trial procedure and environmental release is also considered, which includes laboratory experiments, animal feeding studies, and study of potential environmental. The goal is to discover whether the test plant is safe for consumption and does not pose any serious dangers to the environment or human health. The regulatory data are generated in the laboratory, greenhouse, and field experiments during the assessment cycle from the biosafety regulatory platform. Upon the satisfaction of all requirements and minimal potential of risk, a conditional or unconditional approval of the application is communicated. This is followed by the notification of the applicant, regulatory bodies, and the public. Public involvement in this process ensures transparency regarding the risk assessment process and decision‐making. The notification may accord a time frame for comments on the newly approved product; however, in cases of rejection the applicant may appeal based on approved guidelines. Public involvement involves communication through various means including newspapers, television announcements, social media, and radio.

Environmental release of GMF is an important part of approval of an application, and certain guidelines have been established to regulate the trial of GMF and the environmental release for health and safety. This process starts with a regulated trial process beginning with the modification of a gene and product discovery and development using biotechnological tools. In crops, these traits are usually incorporated into varieties used for transformation rather than commercially viable varieties. Product development is typically conducted in confinement usually in laboratories and greenhouses followed by field trials, and this is important to verify the expression of the gene within the threshold of expected results (Akinbo et al. 2021). The expression of the gene is reviewed by regulatory bodies and experts to establish its safety for environmental release. Certain biological characteristics are considered: the biology and environment relationship of test plants, including the genetic characteristics, reproduction, origin, biodiversity, pest, diseases, and ecological framework.

Environmental release of an approved GM crop does not necessarily mean commercialization as each application is further subjected to post‐release monitoring. This involves risk assessment regarding the possibility of gene transfer between the test plants and the field site to monitor the emergence of volunteer plants and test site evaluation to monitor the effect of the discarded test plant and the existing plants, the potential dispersal of pollen through the field, the survival of plants on the test field, and the aftermath effect of the test plant on the test environment. However, variety development and seed increase in Africa are regulated by procedures under the Seed Act enforced by the National Performance Trial Committee (NPTC) and the National Variety Release Committee (NVRC) (Masehela and Barros 2023). It is important to note that the approval procedures can differ significantly between African countries due to variations in regulatory frameworks, resources, and political considerations. Some countries may have well‐established and transparent approval processes, while others might still be developing their regulatory systems for genetically modified organisms (GMOs).

In Nigeria, the National Biosafety Management Agency (NBMA) is saddled with the sole responsibility of investigating and approving GM foods for commercialization, farming, and consumption. The recent approval of Tela Maize by the Nigerian government raised some eyebrows regarding safety and long‐term effects but the Director General of NBMA Dr. Asagbra enthused that the GM assessment process involved scientific and experimental data analysis, emphasizing that the grain was analyzed for allergenicity, toxicity, and unintended side effects that may result from genetic modification to assess the safety of the GMF in question (Premium times, 2024).

## 7 Regulations and Governing of GMF in Africa

The adoption of GM foods in Africa is gaining attention and has necessitated the establishment of regulatory frameworks that ensure monitoring, risk assessment, and political interference and address public concerns regarding GMF. Many African countries have enacted domestic regulatory guidelines and established biosafety standardized regulatory guidelines through their governmental decision‐making bodies that enable the cultivation and commercialization of GM crops according to the United Nations Cartagena Protocol on Biosafety and the Convention on Biological Diversity Guidelines which is largely focused on risk assessment as GM crops has raised concerns over the years as it relates to the safety of health and the environment (Akinbo et al. 2021). Benin Republic, Eswatini, Ivory Coast, Kenya, Liberia, Mali, Nigeria, South Africa, and Zimbabwe have strong regulatory and monitoring guidelines for GM crops, while Algeria, Angola, Botswana, Burkina Faso, Burundi, Cape Verde, Egypt, Gabon, Mauritius, Mozambique, Rwanda, Tanzania, Togo, and Tunisia are reportedly in the process of developing regulatory frameworks (Gbadegesin et al. 2022). Additionally, Burkina Faso, Mali, and Senegal among other African countries also have established laws for labeling and traceability of GM products in the food supply chain (USDA 2023b).

In Nigeria, the Nigerian National Biosafety Management Agency Act was signed into law in April 2015 and established the National Biotechnology Development Agency (NABDA) and the National Biosafety Management Agency (NBMA) as domestic regulatory bodies focused on biotechnology policies and biosafety regulations of GM crops respectively (Caradus 2023; Bassey 2018). In South Africa, the Genetically Modified Organisms (GMOs) Act 1997 was amended in 2006 and regulates the development and use of GMOs in South Africa; however, South Africa adopted the approach that all gene‐edited products be treated as GMOs and be regulated as GMOs (Pillay and Thaldar 2018). Mali adopted the Biosafety Act in 2008 with regulatory bodies that work simultaneously to ensure the safety of GM products for consumption and environmental release. The regulatory consortium includes the National Competent Authority (NCA), the National Biosafety Committee (NBC), the National Focal Point, and the Public Institutional Biosafety Committees (PIBC) (USDA 2023b). Kenya passed the Biosafety Act 2009 into law in 2009 to facilitate and reduce potential risks posed by GM products; this was closely followed by the establishment of the National Biosafety Authority (NBA) for general supervision and control of the development of biotechnological products. NBA works in close relationships with the National Environment Management Authority (NEMA), Kenya Plant Health Inspectorate Service (KEPHIS), Directorate of Veterinary Services (DVS), Department of Public Health (DPH), Kenya Bureau of Standards (KRBS), Kenya Wildlife Services (KWS), Kenya Industrial Property Institute (KIPI), and Pest Control Products Board (PCPB) (USDA 2023a). In the same year, Senegal adopted its first biosafety law act of 2009 and established the National Biosafety Authority (NBA) and National Biosafety Committee (NBC); this act was modified in 2017 splitting the NBA into Orientation Council (OC) and Executive Bureau (EB) and a new regulatory body: the Scientific and Technical Committee (STC). This review mandated OC as an advisory body and authorized EB primarily as a decision‐maker in GM products. Senegal replaced the Biosafety Act of 2009 in 2022, approving a guideline for the commercialization of GM products in Senegal (USDA 2023b). The biosafety law of 2016 in Cote'de Ivoire established the National Biosafety and Biosecurity Commission (CNBIOS) and the National Biosafety Observatory (ONBIOS); however, these are not yet in operation (USDA 2022). In 2012, Burkina Faso adopted a biosafety law and established the National Biosafety Authority (NBA) charged with approving research, importation, and exportation of GM products. This regulatory body was set to function with the National Biosafety Scientific Committee (NBSC) and the National Biosafety Observatory (NBO) (USDA 2023b). Mauritania signed off a biosafety law in 2022, which established the National Biosafety Authority (NBA) and National Scientific Committee for Biosafety (NSCB) tasked with administrative functions related to GM products and scientific recommendations, respectively (USDA 2023b). Niger passed its first biosafety law in 2019, with the National Competent Authority (NCA), in 2021, the National Technical and Scientific Committee on Biosafety (CTSNB) which oversees the use, examination, and proposition of GM products within the country (USDA 2023b).

Across regions, the Economic Community of West African States (ECOWAS) and Common Market for Eastern and Southern Africa (COMESA) have made great efforts to harness biosafety regulations among member states (Akinbo et al. 2021); the Africa Union Development Agency‐New Partnership for Africa Development (AUDA‐NEPAD) altered in 2018 established the Integrated Vector Management (IVM) to build regulatory capacities in favor of scientific researchers to ensure safe development and dispersal of genetically based vector control innovative tools (Savadogo 2022). Benin, Guinea‐Bissau, Cote d'Ivoire, Burkina Faso, Mali, Niger, Togo, Senegal, Cape Verde, Gambia, Ghana, Liberia, Nigeria, and Sierra Leone are ECOWAS member states under the ECOWAS Biosafety Act which allows the movement of GM products across member states; it also allows a formal recognition other safety assessment of member states as equivalent. The law establishes the Regional Biosafety Authority (RBA) which is a framework of the ECOWAS Commission, the Regional Biosafety Committee (RBC), and the Regional Scientific and Technical Biosafety Committee (RSTBC) (USDA 2023b). International organizations such as the World Health Organization (WHO), Organization for Economic Cooperation and Development (OECD), Food and Agriculture Organization (FAO), and Codex Alimentarius Commission have played significant roles in the regulation of GMF in Africa (Gbadegesin et al. 2022; OECD 2021; WHO 2001). PPP has also been involved in the quality regulation of GMF in Africa.

## 8 Consumer Perception and Ethical Concerns of GMF in Africa

GMF has raised dramatic debates and public concerns over the safety and sustainability of food. Public attitudes and perceptions toward GMF are crucial as they influence government regulation, consumer consumption, and adoption in food production (Mnaranara, Zhang, and Wang 2017). Major pointers of concern are largely around environmental risk and health hazards. Culture and belief, religion, cost, and lack of correct knowledge have also raised concerns.

Dexter et al. (2019) reported a cross‐sectional study on the knowledge, attitudes, and perception toward GMF in Zimbabwe, 60% of the study population showed a poor level of knowledge of GMF with 38% having a negative perception toward GMF. Seventy percent associated increased health risk to GMF, and 72% believed that profit maximization is the major drive for GMF introduction into the food production industry. This is similar to a cross‐sectional quantitative study in Kampala City, Uganda. 45.3% of study participants associated GMF with food safety risks, and 44.3% perceived them to be toxic to human health and the environment. The acceptability of GMF in the study area was largely associated with gender, education level, nutritional value, and health effects (Nowamukama 2022). Mustafa et al. (2023), in a similar study, assessed the level of knowledge of GMF among respondents in Uganda; 31.9% and 74.7% claimed to have heard of GMF and indicated that they had moderate–high understanding of GMF, respectively. Furthermore, 86.8% of those with moderate to high knowledge of GMF associated their knowledge to stream media outlets. 62.3% said that GMF is unhealthy, and 30.8% were indifferent about it. Additionally, 37.7% showed concern about the potential emergence of super pests. Both studies in Uganda reveal a negative perception of GMF which is not necessarily based on the knowledge of GMF but an in‐depth understanding of the role of GMF in Africa's food industry and regulation put in place to mitigate potential risk. The perception of GMF in Kenya differs slightly from reports in Uganda as Kunyanga, Mugiira, and Muchiri (2024) reported 65% of respondents acknowledged that GMF increases the global food supply, makes food affordable (57%), reduces problems of pests (89%), and produces foods with high nutritional value (50%). Notably, 63% believe that GMF is safe and a solution to Africa's food insecurity. However, 36% of respondents associated GMF with health hazards, environmental risk, and biodiversity loss (32%). A comparative insight between the above study in Kenya and a previous study reported in 2004 shows a similar consumer perception in Kenya's population, as over 80% of study respondents acknowledged the increased productivity in food supply by GMF, with only 36% being concerned about health and safety of GMF foods on consumers (Kimenju et al. 2004). Kunyanga, Mugiira, and Muchiri (2024), in a recent study, reported over half of study participants indicated that GMF is safe and over 90% indicated that there is a low threat of GMF on the environment, human health, and animal health. Understandably, the relationship between GMF health concerns and GM food as safe is moderately negative (Kunyanga, Mugiira, and Muchiri 2024), and studies indicate a relatively significant level of acceptance in Kenya.

A report in Abuja, Nigeria, on consumers' perception of GMF showed over half of the respondents claimed no prior knowledge of GMF side effects, however, and believed that long‐term consumption may affect health and environmental safety. A significant number of respondents in this study reported by Iroh et al. (2021) are willing to purchase GMF based on nutritional value, environmental benefits, and low cost of GM foods. 66.7% of the respondents agreed that their decision to purchase GMF is majorly influenced by cost. In summary, this study showed that respondent's willingness to buy GMF is primarily influenced by affordability, despite 67.46% of respondents perceiving GMF as harmful to human health. A weak correlation (*r* = 0.284, *n* = 252, *p* < 0.001) was observed between the perception of harmful to human health and willingness to buy GMF (Iroh et al. 2021). A comparative analysis between reports of two studies reported in Enugu, Nigeria, in 2016 and 2023 showed that 58.3% of respondents will consume GMF in 2016 and 39.5% will consume GMF in 2023. However, in both studies, respondents agreed that consumption of GMF could be harmful to human health as both groups of respondents shared a similar view of GMF being artificial, and this perception may have been muddled by the low knowledge of GMF in both studies (Anugwa et al. 2023; Eneh 2016). The attribute correlation between a low level of knowledge and negative perception of GMF is further strengthened based on the study by Egbe, Bukar, and Adebayo (2019) in Kaduna, Nigeria. Reports showed 86% of the study population showed a high level of GMF knowledge with 70.9% and 81.6% degree and post‐degree holders, respectively. Among this sect, a good perception was observed as they believed that the standard of living would be improved and were willing to consume GMF. A group of people relied on religious leaders for affirmation around GMF. Obi‐Egbedi, Ogungbite, and Oluwatayo (2020) also reported the high influence of education and awareness on the perception of GMF among farmers and consumers. Research carried out by Popoola et al. (2017) on the willingness to consumption of GMF by Nigerians showed that age and socioeconomic status of consumers may affect purchase decisions. Respondents also raised concerns about the threats of GMF to climatic change and environmental pollution but are considerably willing to purchase GMF if the advantages supersede the limitations posed by GMF products. Furthermore report assessment of economic impact of Bt Cowpea in the Middle‐belt, South–South, and Northern geopolitical zones of Nigeria showed a positive perception toward Bt Cowpea as producers and consumers alike perceived it to be a healthier option (Gbegbelegbe et al. 2015). Regarding farmers in Nigeria, Oparinde et al. (2017) assessed farmers' views on the cultivation of provitamin A GM cassava, in Oyo and Benue state, Nigeria. A positive perception was observed in the two states as farmers showed high intention to cultivate GM cassava largely based on the nutritional benefit and low fertilizer requirements. Additionally, smallholder farmers in rural areas in Nigeria largely consume food produced locally from their farms. This can be considered consumers' and farmers' perceptions.

In KwaZulu‐Natal, South Africa, Van Zuydam, Kempen, and Truter (2023) reported a study on South African Consumer's knowledge of GMF products and influences that affect the purchasing decision of GMF. Results showed 87% of the study participants know GMF, 66% stated that the cultivation of GMF will meet good demand of the population, and 56% agreed that pesticide input is inversely proportional to GM technology; however, 52% associated allergies and cancer to the consumption of GMF, and 51% agreed that the cultivation of GMF possesses a level of environmental risk. Similar studies were reported in Nigeria, and 47% of respondents agreed that the low price and increased nutritional value of GMF will influence their purchasing decisions. Although the demographic impact was not within the scope of the study, Van Zuydam, Kempen, and Truter (2023) suggest a probable underlying influence of level of education on consumer perception of GMF. In a similar study in Cape Town, a choice experiment approach was used to examine consumer preferences for GM organism (GMO) food products, which showed customer reluctance in consumption (Ntuli and Dovey 2020). Another study reported by Gastrow et al. (2018) on public perception of biotechnology in South Africa showed about half of the study population (54%) is aware that the cultivation of GMF in the country is approved. Forty‐nine percent believe that GMF is safe for consumption with 45% of the study application believing that GMF has a higher environmental cost relative to traditional farming methods. Fifty‐three percent attribute GMF to increased economic impact. Additionally, an increased number of respondents' purchasing decisions of GMF increased from 50% to 77% upon further consideration of health safety, 51%–77% on cost considerations, and 51%–68% on environmental considerations. Forty‐one percent agreed that GMF infected with the plan of GMF suggesting that religious belief plays a role in acceptance of GMF. Age and level of education were found to play a role in GMF acceptance. A total of 44% of respondents felt that GM foods were effectively regulated by the government.

In Ghana, Owusu‐Gyan, Achiaa, and Chrislie (2023) studied the effect of objective knowledge on consumer acceptance toward GMF in Ghana, and results show that only 10.61% agreed that GMF showed a possibility to affect the genetic makeup of the consumer, 85.27% believed that GMF does not have reduced nutritional benefit, and 84.09% believe that GMF does not pose environmental risk. 83.10% of the study population believes that GMF is important to solving problems related to food insecurities. However, the level of education was observed to impact positive consumer perception of GMF. In a recent study in Ghana, Ogwu et al. (2024) reported that safety concern is the major driver for accepting or rejecting GMF and further suggested that the acceptance or rejection of GM crops is reaped to the religious beliefs of individuals; additionally, findings from this study implicate the yield and taste of GMF as a driver for acceptance of GMF in Ghana. Nevertheless, education is reportedly relevant in preventing negative economic, ecological, and human health consequences in the growth of GMF in Ghana. The knowledge and understanding of GMF among smallholder farmers in Northern Ghana were reported by Zakaria et al. (2022), and 82% of farmers showed a level of knowledge about GMF at different depths. Analysis of respondents' narratives revealed different views of GMF ranging from fictitious to factual knowledge. 17.5% of the respondents gave accurate information about GMF. This study reveals that knowing GMF does not improve consumer perception; however, a correct and high level of knowledge on GMF will go a long way in improving acceptance of GMF.

Consumer perception of GMF in Africa is largely influenced by the level of consumer education, safety concerns, environmental risk, culture, and religious beliefs of Africans. Most people view GMF as artificial and hence tag all GMF products as unsafe as they believe it poses a level of risk to health and the environment; some consider this belief as regard one‐off consumption, while some believe it poses a risk based on length of use. Culture and belief have also been implicated in influences of negative consumer perception toward GMF, as some believe that GMF is mystic, fictitious, or associated with witchcraft, while another sect believes it is outside the plan of God, which goes to further emphasize that GMFs are unnatural (Kamau and Karitu 2023). Furthermore, the perception of risk it poses to the environment has been reported in different regions of different countries in Africa as it is believed to distrust the natural ecological cycle. Nevertheless, all influencers of the negative perception of GMF are largely dependent on the level of education and underlying socioeconomic conditions of individuals as the cost, age, nutritional value, and food insecurity considerations affect perception. The level of knowledge has been established as a major factor affecting consumer perception; however, the source and quality of the knowledge have proved to be relevant as well because previous reports have shown that most consumers have incorrect information and misconceptions about GMF. Inclusive programs and farmers training on the approved utilization of GM technology are essential to avoid adverse effects on the environment. Additionally, government programs and interventions should utilise public resources and channels to convey information about legally cultivated GM crops and enforce labeling of GMF products to enable consumer‐based choices. As with the Tela Maize, consumer perception and concerns raised are the seed being patent‐locked and lacking reproducibility after the first generation, planting along with glyphosate may cause loss of soil fertility and inability to use the farm or soil for other crops, signed policy with biotech company violating Nigeria food sovereignty, and the purported yield of 10 tons per hectare under good agronomic condition according to Africa Agricultural Technology Foundation (AATF) as against 2.2 tons yield by previous strain is untrue (Centre for Food Safety and Agriculture Research—CEFSAR).

## 9 Recommendation

The emergence of GMF has posed a significant debate, especially as it has to do with the environmental risk, toxicity, health threat, and the long‐term effect of the technology and processes involved. Although there has been no evidence of GMF being directly linked to death or environmental degradation, these claims should not be looked over or undermined.

The associated environmental risk mostly engaged is in line with the disruption of the nominal ecological flow, gene transfer, and biodiversity as well as weed and pest resistance. Biosafety methods should be adopted by scientists. Adoption of biotechnology should also be followed by stringent methods that assess the effect of a test plant on the test environment, its effect on surrounding plants and animals, and ecological analysis on plant nutrients to assess the ecological framework of the environment at a given time. Furthermore, sustainable processes should be used in all processes of GMF development to improve perception and reduce possible pollution in the future. Additionally, GM technology should not replace conventional farming practices, mixed practices should be encouraged to enable consumer choice and reduce the possibility of continual use of GMF, whose long‐term effect has not been thoroughly assessed, and a mixed approach will also reduce the possibility of pest and disease evolution that introduces resistance and supersedes GM technology. Environmental regulatory bodies should be consulted and stationed at every level of product development to enforce and advice on environment‐related laws and sustainability.

Health‐related risks include antibiotic resistance transfer, toxicity, allergic reactions, unscientific claims of cancer risk, and general food safety concerns. Based on consumer perceptions in Africa, people believe that consumption of GMF increases the risk of health‐related illnesses such as cancer, toxicity, and transfer of antimicrobial resistance (AMR) thereby elevating the global burden of AMR in the healthcare system and allergies. Consumer choice is a major solution to the perception of health‐related risk, as this affords every consumer the right to choose what they consume based on their health requirements. Food safety measures from farm to fork should be adopted. As a food safety concern, manufacturers' poor labeling of products allows the consumption of GMF by consumers with a lack of information on its content. Traceability and accurate labeling of food products should be strictly enforced to create trust and transparency. The approval of cultivation, commercialization, importation, and exportation of GMF products have been criticized by a sect of Africans that believes GMF is a profit‐making scheme based on the lack of trust in the government and food production industry. Consumer choice will give farmers and the government the opportunity for improvement in the adoption of GMF as a significant backlog is the slow pace of adoption and total ban of GMF in some African countries due to concerns.

Additionally, the long‐term effect of GMF should be studied over several years to carefully evaluate its prolonged effect on test sites, consumers, the environment, health systems, and the surrounding plant and animal life. Today the African consumer has a huge distrust for GMF choosing rather to see it as a weapon through which the Western world is likely pushing for a reduced world population as possibly seen with COVID‐19 vaccines. Transparency in research and documentation culminating in the production of these GMFs should be made available online with a detailed process flow to gain public trust.

## 10 Conclusion

The ever‐growing population in Africa, with the associated food demand and the problems of food production in the continent due to climate change, infrastructure, pests, and diseases, has led to the shortage of food, acute hunger crises, and the progression of food insecurity. The problem of food insecurity has largely affected the lives of Africans in terms of health, environment, and socioeconomic development. GMFs hold a significant potential to support environmental practices, global economic sustainability, and food security due to their ability to boost crop output and decrease the use of agrochemicals. Although Africa bears the heaviest burden of food insecurity, there has been poor adoption of GMF programs in the region due to uncertainty of GM technology regarding food safety, health, and environmental risk and toxicity that has greatly affected consumers' and farmers' perceptions.

Consumers' perception of GMF in Africa has been relatively low regardless of the approval and intervention of government agencies, PPPs, NGOs, and regulatory bodies. The negative perception is largely due to very limited knowledge and wrong knowledge of GMF products among people which manifests in a lack of interest to purchase or consume GMF. The demand for sustainable, environmentally‐ and health‐friendly features are the most important factors that affect the African consumers' purchasing decision of GM food products. The intention to consume GMF is largely influenced by people's knowledge and perception of GM technology, which is further influenced by cost, nutritional value, taste, religion, culture, and socioeconomic factors as underlying factors. There is a need to improve people's awareness and knowledge of genetic engineering processes in food production so that they make informed dietary food choices. Negative attitudes toward genetically modified foods influence low desire to consume genetically modified foods among Africans.

Aside from increasing the level of knowledge among Africans, consumers need to be re‐educated about GMF as some people do know GMF, but their knowledge is solely a product of misinformation and lack of awareness on the issue of food insecurity that GMF can solve. Proper information through media channels and labels of food products on the value of GMF may trigger interest as well as informed identification and decisions about GMF products on the market, thereby benefitting from GMF. Improved knowledge will further assist consumers in dismissing misconceptions that have developed because of the inability to rectify unscientific claims related to GM technology. Scientists must inform decision‐makers so that nations can make autonomous decisions about biosafety and successfully approve GMF and crops for judicious use in Africa.

## Author Contributions

**Adeola Omotosha Dolapo:** conceptualization (equal), writing – original draft (equal). **Helen Onyeaka:** conceptualization (equal), project administration (equal), writing – original draft (equal). **Chiemerie Theresa Ekwueme:** data curation (equal), writing – review and editing (equal). **Esther Ibe Njoagwuani:** data curation (equal), writing – review and editing (equal). **Olaoluwa Olowe Ayomikun:** data curation (equal), writing – review and editing (equal). **Chidinma Ezinne Ngene:** data curation (equal), investigation (equal). **Comfort Adeola Olatunji:** data curation (equal), writing – review and editing (equal). **Iyiola Oladunjoye:** data curation (equal), writing – review and editing (equal). **Ifeanyi Mazi Michael:** data curation (equal), writing – review and editing (equal). **Hope Akegbe:** data curation (equal), writing – review and editing (equal). **Phemelo Tamasiga:** conceptualization (equal), writing – original draft (equal). **Soumya Ghosh:** conceptualization (equal), writing – original draft (equal).

## Conflicts of Interest

The authors declare no conflicts of interest.

## Data Availability

The authors have nothing to report.
